# Bell—Croup

**Published:** 1881-08

**Authors:** Walter M. James

**Affiliations:** Philadelphia


### BELL.—CROUP.

WALTER M. JAMES, M. D., PHILADELPHIA.

AT 3 o'clock on the morning of March 23d, 1881, was called to see a little boy three years old, suffering from croup. When I entered the room, he was gasping for breath, throwing himself wildly about, kicking and crying, as loudly as the state of his larynx would permit, for "more air in his mouth."

His cough was *barking*, resembling the deep baying of a watchdog. Tongue *red on edges, whitish in centre with raised papillæ.* I gave *Bell.* 2 c., in water, a dose every ten minutes. In ten minutes, his breathing became easier. In one hour and twenty minutes, he was breathing as noiselessly, as though nothing were the matter. At nine o'clock in the morning I called again, and found him perfectly well.

